# Imaging gastric cancer metastasis progression in an organotypic, three-dimensional functional model of the human peritoneum

**DOI:** 10.1515/pp-2024-0020

**Published:** 2025-03-06

**Authors:** Arianna Castagna, Frank-Jürgen Weinreich, Andreas Brandl, Janine Spreuer, Nicola Herold, Birgit Schittek, Marc André Reymond, Wiebke Solass

**Affiliations:** Department of General and Transplant Surgery, University Hospital Heidelberg, Heidelberg, Germany; Department of General and Transplant Surgery, University Hospital Tübingen, Tübingen, Germany; Section for Clinical Bioinformatics, Department of Internal Medicine I, University Hospital Tübingen, Tübingen, Germany; Department of Dermatology, University Hospital Tübingen, Tübingen, Germany; National Center for Pleura and Peritoneum, University Hospital Tübingen, Tübingen, Germany; Institute of Tissue Medicine and Pathology ITMP, University Bern, Niederscherli, Switzerland

**Keywords:** gastric cancer, metastasis progression, organotypic model, cell culture imaging

## Abstract

**Objectives:**

Despite the introduction of multimodal treatment regimens, the prognosis of gastric cancer peritoneal metastasis (GCPM) remains poor. To establish efficient therapies, a deeper understanding of pathophysiological mechanisms in the development of GCPM is necessary and this requires adequate functional models. Therefore, we established a three-dimensional model to study tumor adhesion, invasion and growth.

**Methods:**

A co-culture of peritoneal mesothelial cells with fibroblasts and collagen I was cultivated to further seed human gastric cancer cell lines on the surface. Different imaging techniques (optical microscopy, immunohistochemistry, scanning (SEM) and transmission (TEM) electron microscopy) served as tools to proof the sustainability of the model.

**Results:**

We demonstrated the feasibility of creating a robust GCPM model. We showed that the model is reproducible under various conditions (6-, 12-, and 24-wells) and pre-analytical processing is possible. The imaging was feasible and allowed the comparison of morphological changes on the GCPM model to normal human peritoneum.

**Conclusions:**

We established a reproducible and robust organotypic model of GCPM which can be used to generate deeper knowledge on the pathophysiology of GCPM and might serve as a platform for testing different chemotherapy schemes in order to establish a personalized treatment for patients with GCPM.

## Introduction

The majority of patients diagnosed with gastric cancer (GC) present in an advanced or metastatic tumor stage, in which liver and peritoneum are the most frequent metastatic sites [1], 2]. Metastatic GC is associated with a poor prognosis, and although multimodal and especially targeted therapy regimens are on the rise, the median overall survival remains 7–9 months [3].

Peritoneal metastasis of Gastric Cancer (GCPM) represents one key factor in the patient journey with GC, as approximatively 40 % develop peritoneal metastasis during their disease. While systemic therapy or multimodal therapy underwent significant improvements in overall survival and quality of life, especially due to a docetaxel-based chemotherapy combination consisting of fluorouracil, leucovorin, oxaliplatin, and docetaxel (FLOT) [4], as well as targeted therapy or checkpoint inhibitors therapy (Her2, PD-L1), the early detection of peritoneal metastasis (PM) or the prevention has not been successful so far [5], 6]. Better knowledge of the pathogenesis of PM is needed for improving patient care: focusing on the pathogenesis and on the molecular development of the peritoneal foci may permit an earlier detection of peritoneal tumor progress, as well as new therapy targets.

The pathogenesis of PM is a multistep process requiring adaptation of both the tumor cells and the tumor microenvironment. Underlying mechanisms of interplay between free tumor cells shed into the peritoneal cavity, and PMCs, as well as the role of their interactions in cancer progression are not yet fully understood. Starting from the individual cancer cell, stages of progression include cell shedding from the primary tumor, transcoelomic transport, adhesion to the peritoneum, migration, invasion, proliferation, and epithelial-mesenchymal transition [7].

The current range of cancer models, including two-dimensional (2D) models and organoids, offer distinct advantages but also face notable limitations. 2D models are commonly utilised due to their affordability and simplicity; however, they are unable to fully reflect the intricacies of tumor biology and frequently lack clinical applicability [8]. Promising PM-derived organoid cultures have been established during the last years, highlighting numerous advantages [9], holding also limitations such as the tumor microenvironment. Indeed, organoids often lack critical components, such as stromal and immune cells, which are essential for accurately modelling tumor behaviour [10]. Moreover, the establishment of peritoneal metastasis models is a more intricate process than that of traditional models, as it necessitates the replication of both tumor growth at the implantation site and metastatic dissemination.

Reconstructing a 3D-human peritoneum model might overcome this shortcoming and will permit to observe and describe the adhesion and invasion patterns of various tumor phenotypes into the peritoneum at various evolution stages. Most models though are xenograft models in immunocompromised animals, largely preventing immunological studies [11], [12], [13]. While PDX models offer a more accurate representation of tumor heterogeneity and genetics, their development is time-consuming, costly and poorly suited to align with all real-time clinical needs [14]. Further, alternatives for animal models are urgently needed.

Building upon the prior art [15], 16], we established an organotypic 3D reconstruction of the human peritoneum.

## Materials and methods

### Study design

We established a 3D reconstruction of the human peritoneum mimicking the metastatic process of GCPM by using commercially available cell lines. The morphology and structure of the 3D model were analyzed by means of light microscopy, scanning electron microscopy (SEM), and transmission electron microscopy (TEM). Gastric cancer cell lines were seeded on the surface of the mesothelial cell layer, and their metastasis behavior was characterized over time.

### Ethical and regulatory background

This study was approved by the Ethics Committee of the University of Tübingen on March 3rd, 2020 (Reference number 123/2020BO20).

### Cell culture and reagents

Collagen type-I from rat tail was provided from Corning (Discovery Labware Inc., USA). Normal human dermal fibroblasts (NHDF) were obtained from Bioproducts Boehringer Ingelheim (Leimen, Germany). Adult human mesothelial cells (AMC) were purchased from Zen-Bio, Inc. (Research Triangle Park, NC). The AMCs were isolated from human omental adipose tissue from consented adult donors undergoing elective gastric bypass surgery. The cell line was cultured from passage 3 to passage 9 for maximum cell number, whereas experiments were done at passage 5. Cell lines were grown to 90–100 % confluence for experimental usage: in detail, NHDF cells were cultured in 150 cm^2^ culture flasks (Falcon, Corning, New York, USA) in Dulbecco’s Modified Eagle Medium (DMEM) + GlutaMax-I (4.5 g/L d-glucose), containing 10 % fetal bovine serum (FBS), 100 U/ml penicillin G and 100 μg/mL streptomycin at 37 °C and 5 % CO_2_ in humidified incubators. AMCs were also grown in 150 cm^2^ flasks at the same conditions except Medium M 199 (Zen-Bio Inc Research Triangle Park, NC) instead of DMEM as recommended by Zen-Bio company. All cell culture solutions were obtained from Gibco/Life Technologies GmbH, Darmstadt, Germany. Trypsin/EDTA (0.05 %/0.02 %) was used for detachment. Multi-layered 3D tissue model was established in 6, 12, and 24 well plates (Falcon, Corning, New York, USA) containing hanging Millicell^®^ Cell Culture Inserts with polyethylentherephtalat bottom mesh (pore size of 0.4 µm) used as a model basement. The experiments were then repeated with immortalized mesothelial cells available on the market (MeT-5A). MeT-5A were purchased from American Type Culture Collection (ATCC^®^ CRL-9444™, LGC Standards, Germany) and cultured in Medium RPMI 1640+GlutaMax™-I (Invitrogen) culture medium supplemented with 10 % fetal bovine serum, 100 U/mL penicillin G and 100 μg/mL streptomycin.

### Cancer cell lines

Two human gastric adenocarcinoma cell lines MKN-45 and 23132/87 (German Collection of Microorganisms and Cell Cultures Braunschweig, Germany) were seeded in 150 cm^2^ culture flasks (Falcon, Heidelberg, Germany) and maintained in RPMI 1640+GlutaMax™-I (Invitrogen) culture medium supplemented with 10 % fetal bovine serum, 100 U/mL penicillin G and 100 μg/mL streptomycin. The cell lines were authenticated by STR profile analysis (PowerPlex 21HS, Promega), with frozen aliquots from same passages used for experiments.

### Construction of artificial human peritoneal tissue (AHPT)

The 3D reconstruction was established on a Collagen-I basis layer coating on top of a porous insert membrane. After trypsinization of NHDF cells the cell suspension was transferred to a 2 mL polyethylene vial and shook in an overhead rotator until cell counting at low speed. 100 µL of the cell suspension was diluted with 10 mL of CASYton (Schärfe System) and then analyzed with a CASY™ automated cell counting system. NHDF cell suspension containing DMEM + GlutaMax-I (4.5 g/L d-glucose), 10 % fetal bovine serum (FBS), 100 U/mL penicillin G, and 100 μg/mL streptomycin, was seeded on the collagen layer and cell proliferation was allowed for 5 days. Finally, AMC or MeT-5A cells were seeded using the same procedure on the surface in a cell suspension of medium 199 or RPMI 1640 + GlutaMax™-I 10 % FBS. The model was left to grow for 24 h and analyzed. The medium was changed every third day. After tumor seeding, the metastatic process was studied at 6, 12, 24, 36, 72 and 96 h.

## Results

### Morphology of the peritoneal reconstruct

**Figure 1:**
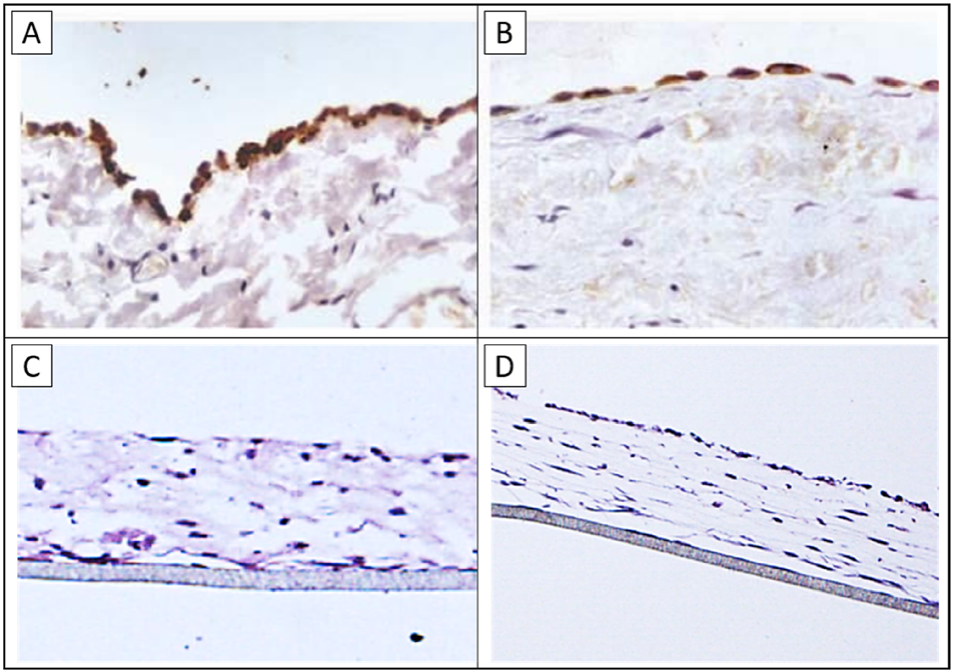
Imaging of peritoneal tissue: (A) and (B) show the morphological structure of the peritoneal surface in a patient undergoing peritoneal dialysis [17]; (C) and (D) representing the peritoneal model reconstructed in our laboratories.

The model was primarily analyzed under optical microscopy (Figure 1): the hematoxylin/eosin evaluations showed a fibroblast and collagen layer on the 4 µm thick, porous supporting membrane. Furthermore, an AMC and MeT-5A monolayer was to be recognized above the fibroblasts. BerEp4 stunning showed the MKN-45 on the surface after seeding.

**Figure 2:**
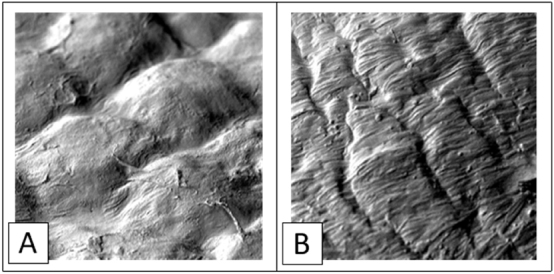
SEM imaging of a surgical omental sample vs. 3D reconstruct’s surface: (A) omental sample after bariatric surgery. (B) Surface of the reconstruction showing the fibroblast-like structure of AMC at the late passage step.

The 3D reconstruction was then processed for analysis with an SEM microscope (Figure 2). The superficial morphology could be overlapped with the typical structure of the human omentum. However, considering the morphology of AMC at a late passage, the mesothelial cells happened to take a fibroblast-like form compared to immortalised MeT-5A.

**Figure 3:**
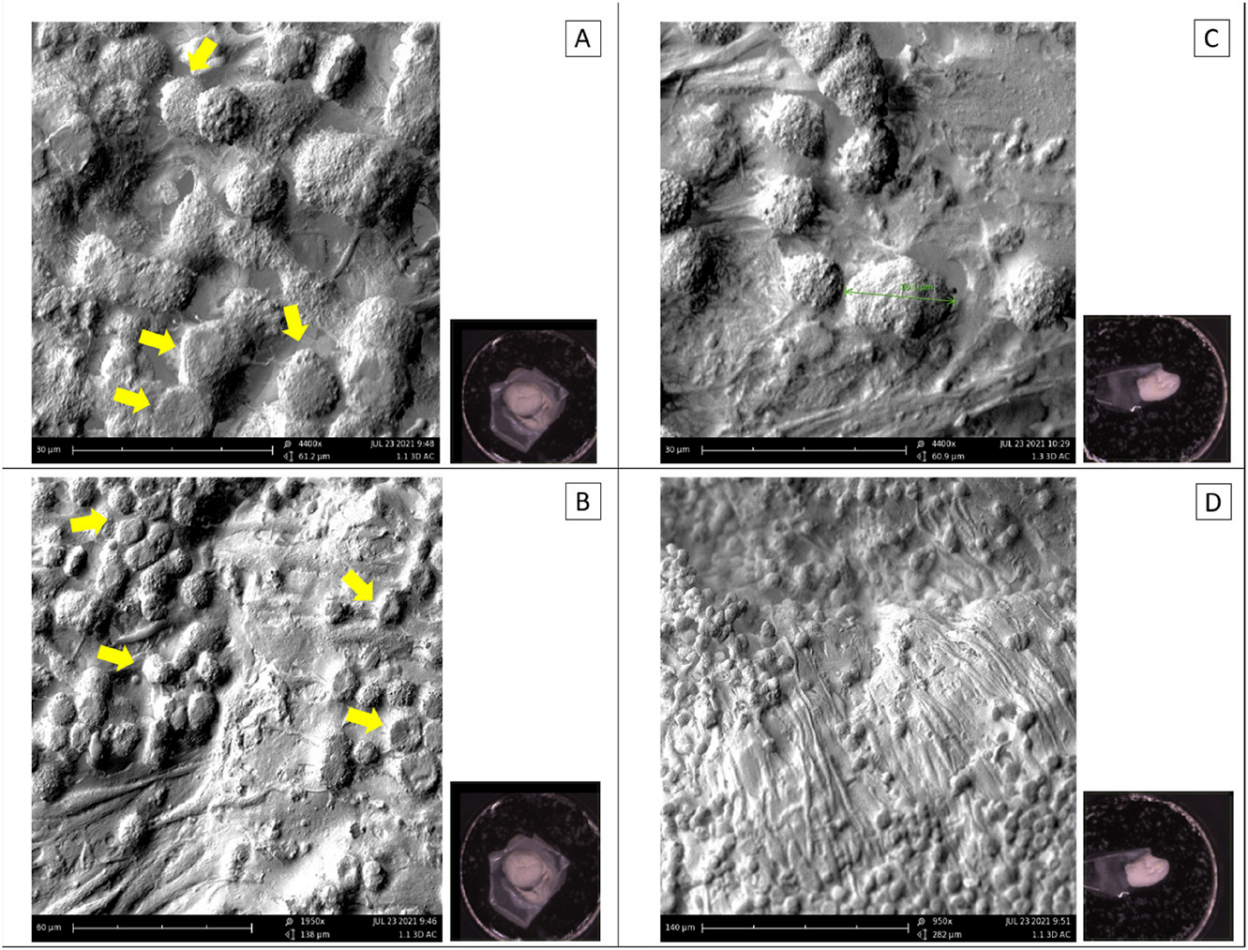
Early vs. late seeding of MKN-45 on the surface of the peritoneal reconstruct: (A) and (B) show how the tumor cells tend to aggregate and flatten onto the mesothelial monolayer after one week on the surface (arrows), if compared to (C) and (D) showing a more preserved morphology of the round MKN-45 (green) only one day after seeding.

Moreover, seeding MKN-45 cells could be followed, also showing a flattering of the tumor cells after longer exposition to the surface (Figure 3).

**Figure 4:**
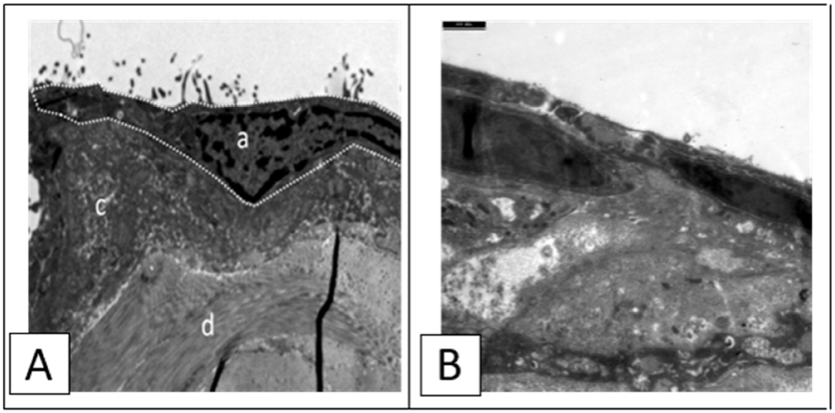
TEM imaging: (A) surgical sample in gastric cancer patient; (B) 3D reconstruct showing the cell-cell junctions and small mocrovillosities.

Further analysis with TEM microscopy highlighted the cell-cell connection pattern, closely similar to the *in vivo* situation (Figure 4). The presence of microvillosities on the surface could also be detected.

### Feasibility of the peritoneal reconstruct

**Figure 5:**
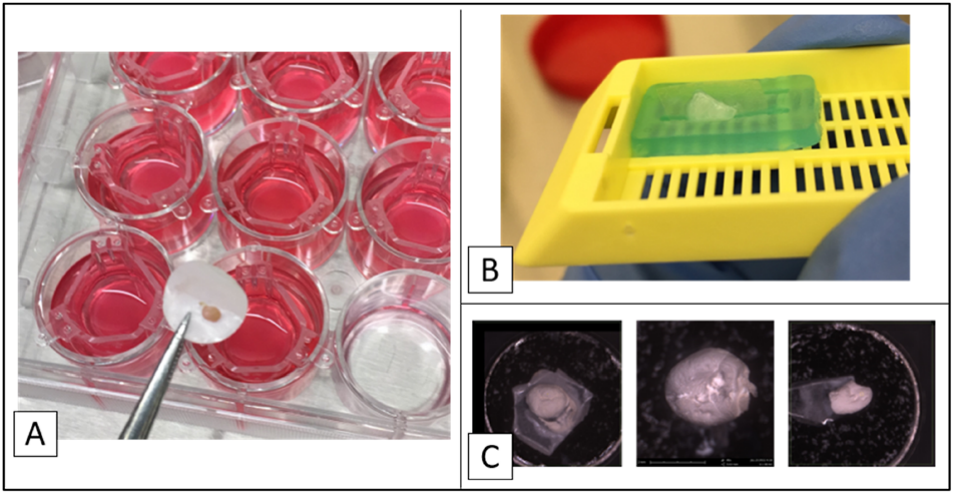
Preanalytical issues: (A) example of 12-well reconstruction on the porous membrane; (B) paraffin embedding process preparation; (C) sequence of 24-well models under SEM microscope.

Culturing the reconstruct was showed to be feasible in 6, 12 and 24-wells (Figure 5). Though producing models of different sizes, the structure and proportional dimensions was maintained. Different preanalytical passages were in all tests possible, though handling bigger 6 wells models offered a much easier cryotome slicing, whereas the small 24-wells reconstruct could be entirely processed, without prior cutting under SEM microscope, allowing the exclusion of a preparation passage and so reducing a preanalytical bias.

### Describing the metastatic process

**Figure 6:**
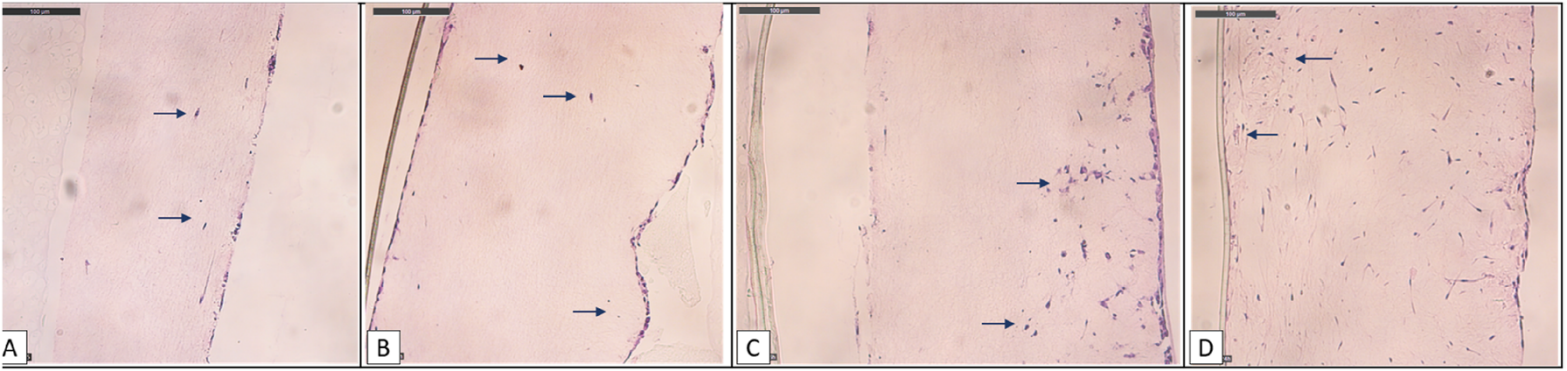
Sequence of the metastatic process in MKN-45, HE – optic microscopy (20×) in the 6-wells reconstruct. (A) 6 h after seeding, only a few single cells infiltrate the fibroblast layer (arrows). (B) 12 h after seeding, MKN-45 shows an increase of proliferation on the surface, with few more single cells penetrating the more profound layers; (C) 24 h growth on the peritoneal model, increasing the cell organization on the surface and begin of a consistent penetration of the fibroblast layers; (D) image of diffuse dissemination of MKN-45 all through the reconstruct, and first small nodes reaching the porous membrane on the bottom.

**Figure 7:**
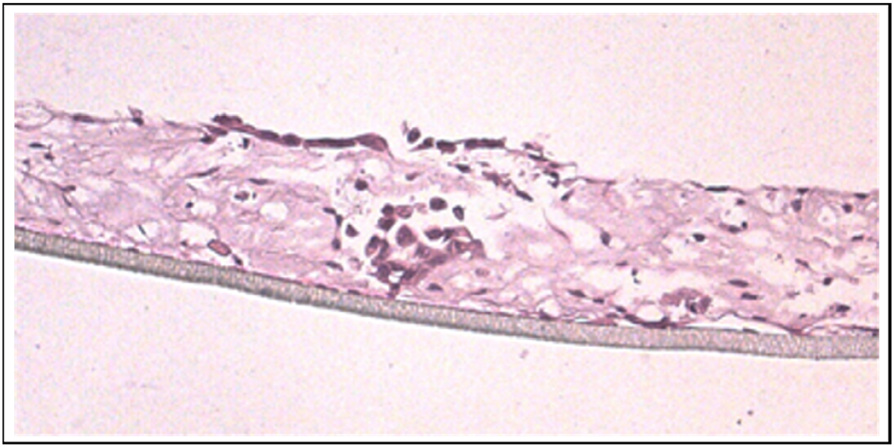
Example of the invasion of a gastric tumor node. MKN-45, 12-wells, Ber-Ep4.

MKN-45 and 23132/87 cells were both feasible to grow on the 3D reconstruction of the human peritoneum. Though, due to their aggressive potential, the MKN-45 showed a quick invasion pattern when compared to the 23132/87. We followed the metastatic process of MKN-45 by analyzing perpendicular slices of the model every 6 h till 96 h after seeding (Figure 6). After only 12 h, the MKN-45 could show an increase in organization (Figure 7), and the first cells began to enter the deeper layers of fibroblasts till the porous membrane was reached (96 h).

23132/87 cell lines showed the same pattern of growth, though they needed a longer exposition to the model. In this case, the full penetration of the model was reached after 1 week.

**Figure 8:**
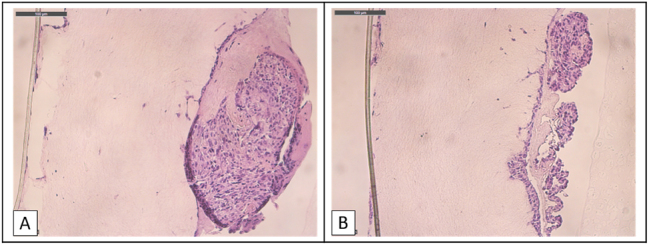
Early aggregate in MKN-45, HE – optic microscopy (20×) in the 6-wells reconstruct. (A) After 36 h seeding the tumor cells aggregate and tend to form a tumor node-like structure. (B) Smaller early aggregate beginning the process of infiltration of the basal membrane.

Moreover, after 36 h seeding of the MKN-45 on the surface, it was already possible to spot previously described major early tumor aggregate on the peritoneal surface [18] (Figure 8).

## Discussion

Efforts to model gastric cancer peritoneal metastasis (GCPM) for a deeper understanding of its pathogenesis and to develop effective treatments have been ongoing. Current models primarily rely on xenografts in immunocompromised animals, 2D cell cultures, cocultures, and organoids. However, more accurate peritoneal models are increasingly needed to overcome diagnostic challenges, unravel the molecular mechanisms of the disease, and explore the tumor microenvironment beyond the limitations of xenografts. While existing models provide valuable insights into tumor behavior on the peritoneal surface, they fail to fully replicate the complex interactions present in the human peritoneum.

This study introduces a novel 3D human peritoneum model that accurately mimics GCPM using commercially available cell lines. Detailed morphological assessments, including light, scanning electron (SEM), and transmission electron microscopy (TEM), validated the model’s accuracy, allowing for morphological characterization of metastatic behavior over time. Notably, the interaction between gastric cancer cells and the mesothelial layer revealed key aspects of metastatic progression, addressing shortcomings of current models.

The study investigated the behavior of fibroblast-collagen layers, AMC/MeT-5A monolayers, and MKN-45/23132/87 gastric cancer cells post-seeding. The model closely mimicked the human omentum, reflecting mesothelial changes and tumor dynamics consistent with *in-vivo* observations. Importantly, it captured invasion patterns and metastatic progression. Comprehensive microscopic analysis validated the model’s fidelity, providing crucial insights into cellular interactions within the peritoneal microenvironment. Its morphological resemblance to the omentum underscores its physiological relevance, making it a valuable tool for studying GCPM and its implications for therapeutic development.

However, the model does not incorporate vascular elements, which limits its ability to simulate and analyze neoangiogenesis, a critical process in tumor growth and metastasis [19]. Without a vascular component, the model cannot fully capture the intricate interactions between the tumor and its microenvironment, particularly those involving endothelial cells and the development of blood vessels. Additionally, understanding the effects of anti-angiogenic therapies – which target the formation of blood vessels – requires a model capable of reproducing the vascular architecture and its dynamic response to tumor growth. Incorporating vascular elements into the model would significantly enhance its utility by enabling a more thorough investigation of angiogenesis, the cross-talk between tumor cells and endothelial cells, and the influence of blood flow on drug delivery and therapeutic efficacy [20].

In the future, we will examine the effects of inflammation and stromal cells on the immunotolerance observed in GCPM progression [21]. Tumor-associated inflammation plays a crucial role in immune evasion and metastasis. By incorporating inflammatory and stromal elements, we aim to better understand how these factors contribute to tumor progression and therapy resistance. Building on colorectal cancer models [22], we will coculture immune cells, particularly macrophages [23], 24] and T-lymphocytes [25], 26], to explore their interaction with the tumor microenvironment. This approach will provide insights into immune evasion mechanisms, informing the development of targeted therapies [27] for GCPM, particularly immunotherapies [28]. Including stromal cells such as fibroblasts [29] will add further complexity, enhancing the model’s relevance and potential for personalized therapeutic strategies.

Incorporating patient-derived cells [30], 31] could further enhance the model’s relevance by increasing heterogeneity, making it more applicable to personalized drug testing and treatment evaluation. Another valuable area to explore is the addition of dynamic fluid flow systems to the 3D model [32], mimicking the fluidic environment of the abdominal cavity. This could provide a more accurate representation of metastatic progression and drug response by introducing mechanical forces into the *in vitro* system.

## Conclusions

This study presents a robust framework, combining accurate 3D reconstruction with detailed microscopic characterization of cellular behavior and invasion patterns over time. The development of a 3D human peritoneum model for GCPM with realistic morphological features represents a significant advancement in the field. This model accurately replicates tumor dynamics, offering valuable insights into invasion patterns and cellular interactions. Our reconstruct of the human peritoneum facilitates the study of peritoneal metastasis by accurately simulating tumor interactions within a realistic peritoneal environment. Indeed, the model incorporates essential cell types, including mesothelial cells and fibroblasts, as well as extracellular matrix components that facilitate the replication of key processes, such as adhesion, invasion, and tumor growth. By capturing these stages in detail, the model may allow the study of the molecular drivers of metastasis and provide a controlled setting for the testing of therapeutic agents, including those targeting adhesion and invasion. However, while it excels in simulating these aspects, there are opportunities for further improvement, for example by co-culturing vascular and immunological components. Future research will focus on incorporating functional assays, establishing standardized validation protocols, and exploring dynamic factors such as immune interactions and fluidic environments, as well as addressing therapy response challenges. Enhancing the model’s accuracy will increase its reliability and potential for personalized drug testing, driving therapeutic advancements for GCPM.
